# Efficacy of Attract-and-Kill Techniques in Controlling *Bactrocera oleae* (Diptera: Tephritidae) in a Highly Variable Olive Production Scenario

**DOI:** 10.3390/insects16111161

**Published:** 2025-11-13

**Authors:** Giacomo Ortis, Giacomo Santoiemma, Federico Marangoni, Francesco Sanna, Maria Rosaria Fidanza, Mario Baldessari, Nicola Mori

**Affiliations:** 1 Department of Biotechnology, University of Verona, Strada le Grazie, 15, 37134 Verona, Italy; federico.marangoni.92@gmail.com; 2 Department of Agronomy, Food, Natural Resources, Animals and Environment, University of Padova, Viale Dell’Università 16, 35020 Legnaro, Italy; giacomo.santoiemma@unipd.it (G.S.); sannino.sanna@gmail.com (F.S.); 3 Research Centre Agricultural Policies and Bioeconomy, Council for Agricultural Research and Economics, Via Barberini 36, 00187 Rome, Italy; mariarosaria.fidanza@crea.gov.it; 4 Technology Transfer Centre, Fondazione Edmund Mach, Via E. Mach 1, 38098 San Michele all’Adige, Italy; mario.baldessari@fmach.it

**Keywords:** olive fruit fly, mass trapping, lure and kill, pest, integrated pest management, semiochemical-based control

## Abstract

The management of the olive fly using sustainable methods includes strategies based on attract-and-kill techniques. Although some studies have shown that lure-and-kill and mass-trapping methods can be effective in certain contexts, their performance under conditions of highly variable olive production remains unclear. In this study, we evaluated the effectiveness of two sustainable control techniques in olive groves located at the northernmost boundary of olive cultivation in Europe. The efficacy of a lure-and-kill product (Spintor^TM^ Fly) and a mass-trapping product (Flypack^®^ Dacus Trap) was assessed over a three-year period by monitoring olive fly population density and infestation levels. We found that the efficacy of attract-and-kill techniques varied over the years. In years of abundant production, the high availability of fruits may reduce the detectability of damage. In contrast, in low production years, the limited number of fruits can lead to higher infestation rates, potentially reducing the effectiveness of the control techniques. Both techniques tested, particularly lure-and-kill, can help maintain low *Bactrocera oleae* population densities. However, they are insufficient to maintain fruit infestation at acceptable levels during years of low olive production, when the adoption of control measures is not economically justified.

## Simple Summary

The olive fruit fly is a major pest in olive cultivation, and its management increasingly relies on sustainable strategies that can reduce the need for insecticide spray coverage. These techniques work by efficiently attracting olive flies to devices that either trap them (e.g., mass trapping) or expose them to toxic substances (e.g., lure-and-kill). The underlying principle is that by removing a significant portion of the adult pest population from the agroecosystem, crop damage can be effectively reduced. Over a three-year period, we evaluated the efficacy of two commercially available products in a region characterized by quantitatively highly variable olive production. The techniques successfully controlled infestation in high-yield years but were insufficient during medium to low production years, when additional control measures are needed.

## 1. Introduction

The olive fruit fly (OLF) *Bactrocera oleae* (Rossi, 1790) (Diptera: Tephritidae) is a common pest infesting olive orchards across the Mediterranean basin, causing both qualitative and quantitative damage to olive production [1,2]. For many decades, infestations of *B. oleae* have been managed with insecticide spray coverage, which has proven effective in reducing pest pressure. However, due to the negative impact on both humans and non-target arthropods [3,4,5], the recent withdrawal of broad-spectrum active ingredients, such as imidacloprid (Regulation (EU) 2019/1090), dimethoate (Regulation (EU) 2020/1643), and phosmet (Regulation (EU) 2022/94), has significantly reduced the number of widely used active ingredients, triggering the evaluation of alternative control strategies [6].

Alternative methodologies for managing *B. oleae* include attract-and-kill methods (AK), biological control, the implementation of sterile insect techniques, and the development of products with repellent and oviposition-deterrent effects [7,8]. In particular, attract-and-kill techniques have received considerable attention, being widely implemented and continuously refined [9,10] due to their lower costs, reduced labor requirements and decreased insecticide inputs [11,12]. All these techniques rely on efficiently attracting olive flies to devices that either retain them (e.g., mass trapping—MT) and/or expose them to toxic substances (e.g., lure-and-kill—LK). The underlying concept is that by removing a significant portion of the adult pest population from the agroecosystem, crop damage can be effectively reduced. Typically, lure-and-kill involves the application of baits containing hydrolyzed proteins mixed with diverse active substances such as spinosad and deltamethrin, while mass trapping employs attractive traps such as McPhail traps and/or cone traps baited with sexual pheromones and kairomones (e.g., ammonium salts) with interior insecticidal surfaces [13,14,15]. However, the efficacy of these methodologies has produced mixed results, likely due to the variability among traps, pest densities, regions, and agroecosystems where the experiments were conducted [14,16,17,18].

In recent years, fluctuations in climatic conditions and olive yields have been reported across Italian regions, leading to substantial production losses [19]. In the Lake Garda area, for example, yields have ranged from 0.5 to 7 tons per hectare [20]. The objective of this study was to evaluate the performance of attract-and-kill techniques in reducing *B. oleae* population density and infestation levels in an olive-growing region characterized by highly variable olive production. Over a three-year period, we evaluated the efficacy of two commercially available formulations: a lure-and-kill (LK) and a mass-trapping (MT) product.

## 2. Materials and Methods

### 2.1. Experimental Orchards

The study was conducted in the seasons 2020, 2021 and 2022 in the Lake Garda area (north-east Italy) on two similar experimental olive groves located in Castelletto di Brenzone (45.688264° 10.753431°, 5.5 ha) and Malcesine (45.778364° 10.820680°, 5.2 ha). The two sites, located 11 km apart, were treated according to the Garda D.O.P. regulations for olive production (Regulation (EU) 2024/1143) and no additional insecticide treatments were performed during the experiments. Olive groves feature the principal varieties of Garda D.O.P., namely Casaliva, Frantoio and Leccino. These olive groves were characterized by trees over 30 years old, trained in a hollow cone system. There were approximately 200 plants per hectare, spaced 5 m apart between rows and 6 m apart within rows. Grass clippings occurred twice a year, once mid-season and once before harvest. No irrigation was applied to the olive groves. The landscape surrounding these sites consisted of other olive groves, urban areas, and woodland patches.

### 2.2. AK Techniques

Two AK techniques were evaluated: LK, a sprayable bait with a mixture of hydrolysed protein as lure and spinosad as insecticide (Spintor^TM^ Fly, Corteva Agriscience Italia s.r.l., Cremona, Italy) and MT, a yellow conic plastic trap containing sexual pheromones and ammonium salts with a transparent cover treated internally with deltamethrine (Flypack^®^ Dacus Trap, SEDQ Healthy Crops, S.L., Barcelona, Spain).

The experimental conditions (e.g., design and layout of the trial) were made according to specific EPPO standards on the evaluation of the efficacy of plant protection products (PP1/108(3) canopy spray and PP1/280(1) bait application).

The LK technique was tested in the experimental site Castelletto di Brenzone, while the MT technique was tested in Malcesine. In each site, an untreated control area of 0.3 hectares was included. Each year, the experiments began in June, during the phenological phase of fruit development [21], when olive fruits become susceptible to *B. oleae* oviposition [22]. This period has traditionally been used in pest management research; however, an earlier deployment is recommended to intercept the spring generations of the pest as well [23]. Bait applications were carried out eight times every 10 days, starting from the stone lignification stage (BBCH 75) and until one week before harvest. The Spintor^TM^ Fly application rate was 1 L of product mixed with 4 L of water per hectare. Pumps and cone nozzles were used to create a spot approximately 40–50 cm in diameter on the foliage of 50% of the plants. In case of heavy rainfall exceeding 20 mm, canopy spray was repeated the following day to ensure continuous protection.

Flypack traps were positioned from the last week of June, with a total of 100 traps per hectare. Since the label states that the attractant lasts 180 days, the Flypack traps were not replaced during the study period.

### 2.3. Efficacy Evaluation

The efficacy of the AK techniques was evaluated based on both olive fly population densities and fruit infestation levels. In addition, the olive production (kg olives/ha) was recorded at the end of the harvest period for each site.

To monitor adult olive fly activity, four yellow roof sticky traps (Bactrap, CBC (Europe) S.r.l.—Biogard division, Grassobbio, Italy; trap sizes 18 × 24 cm) baited with ammonium salts and pheromone lure were placed at each treated plot: one in the center and three along the edges. In the control area, one trap was placed in the middle of the plot. The ammonium salts container was replaced upon exhaustion, while the pheromone vial was replaced every month. The traps were inspected weekly from early June until harvest.

In order to assess the fruit infestation level, olive sampling was carried out every two weeks from stone lignification until harvest (October). On each sampling date, an average of 338 ± 25 SE drupes was collected by sampling olives from all parts of the canopy on approximately 30 randomly selected trees per plot, representing all three varieties. Total infestation was calculated as the sum of olives with eggs, larvae, live or dead pupae, parasitized larvae, exit holes and sterile punctures.

### 2.4. Data Analysis

To test the effect of treatment on olive infestation, generalized linear mixed models with a binomial distribution (logit link function) were built and validated. One model was built for each experimental olive grove. The response variable was the % of infested olives. The explanatory variables were the treatment (i.e., control and Flypack in one grove and control and Spintor Fly in the other grove), the sampling year, and their interaction. The random factor was the sampling date. Pairwise comparisons between treatments within each year were run using Post Hoc tests with Tukey correction of *p*-values. Model residuals appropriately fit the binomial distribution and showed no temporal autocorrelation in the time series. All analyses were conducted in R software (version 4.4.0) [24].

## 3. Results

### 3.1. Olive Fly Population Densities

**Figure 1:**
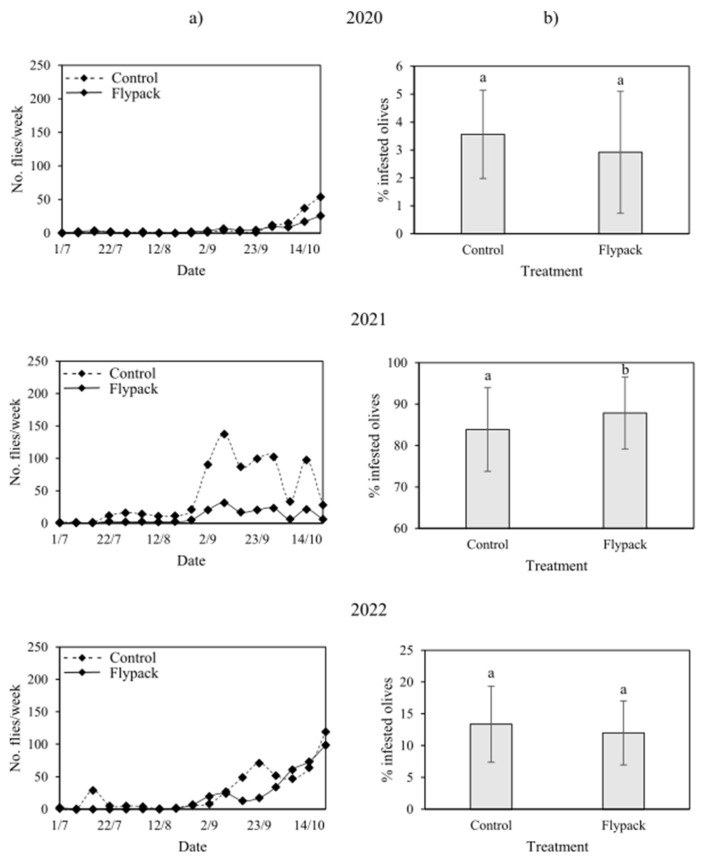
(**a**) Number of *Bactrocera oleae* catches on sticky traps (Bactrap) recorded every 7 days and (**b**) the mean (± SE) number of infested olives at the site where the mass trapping technique (Flypack) was tested over three years. Different letters indicate significant differences among treatments (Tukey’s test: *p* < 0.05).

At the experimental site used for testing MT technique, a total of 90 OLF were captured in the treated plot (6 flies/week) and 133 in the control plot in 2020 (8.3 flies/week). In 2021, 185 OLF were captured in the treated plot (10 flies/week) and 862 in the control plot (45 flies/week). In 2022, the number of OLF captured increased to 354 in the treated plot (22 flies/week) and 851 in the control plot (47 flies/week) (Figure 1).

**Figure 2:**
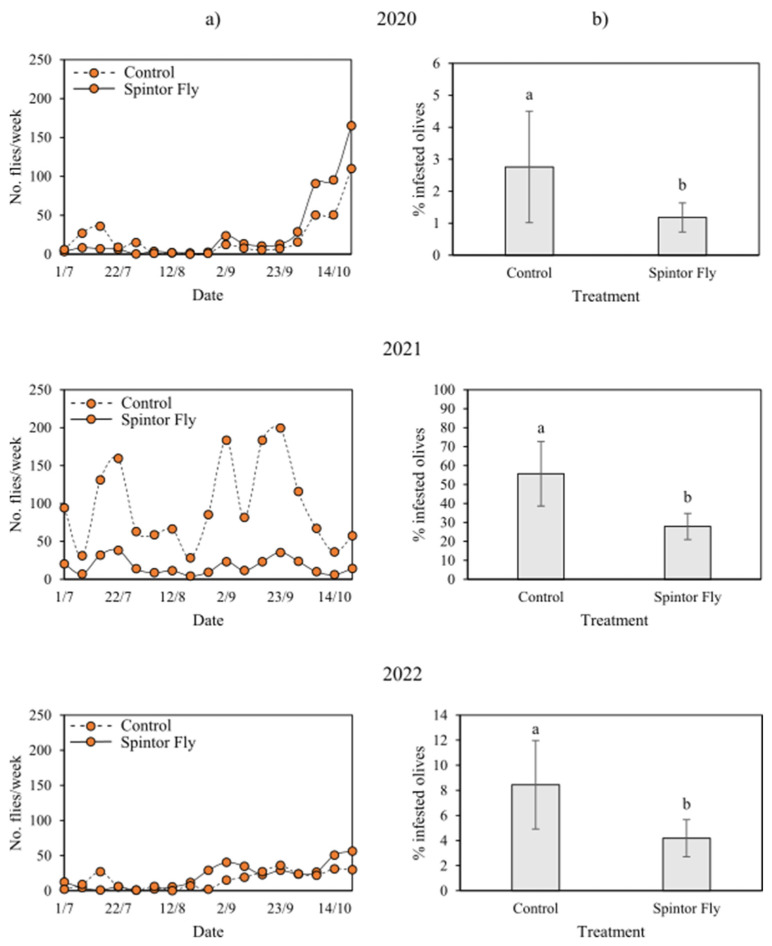
(**a**) Number of *Bactrocera oleae* catches on sticky traps (Bactrap) recorded every 7 days and (**b**) the mean (±SE) number of infested olives at the site where the lure-and-kill technique (Spintor Fly) was tested over three years. Different letters indicate significant differences among treatments (Tukey’s test: *p* < 0.05).

At the experimental site used for testing LK technique, a total of 475 OLF (20 flies/week) were captured in the treated plot and 121 in the control plot in 2020 (7.6 flies/week). In 2021, 367 OLF were captured in the treated plot (17 flies/week) and 1971 in the control plot (94 flies/week). In 2022, the number of OLF captured increased to 408 in the treated plot (21 flies/week) and 375 OLF were captured in the control plot (22 flies/week) (Figure 2).

### 3.2. Fruit Infestation Levels

During the year 2020, mean olive infestation levels were low (<5%) both in treated and control plots and a significant difference was observed between the area treated with Spintor Fly (1.2 ± 0.5%) and control area (2.8 ± 2%), while similar mean infestation rates were recorded in treated (2.9 ± 2%) and control plots (3.6 ± 1%) at the experimental site treated with Flypack. In 2021, an increase in olive infestation rates was detected in the two experimental sites and a significant difference was found between the area treated with Spintor Fly (27.9 ± 7%) and control area (55.7 ± 17%) and the area treated with Flypack (87.9 ± 8%) and control area (83.9 ± 10%). In 2022, similar mean infestation rates were recorded in treated (12 ± 5%) and control plots (13.4 ± 6%) of the experimental site treated with Flypack, while a significant difference was found between treated (4.2 ± 1%) and control plots (8.4 ± 3%) of the experimental site treated with Spintor Fly (Table 1 and Table S1, Figure 1 and Figure 2).

### 3.3. Olive Production

**Figure 3:**
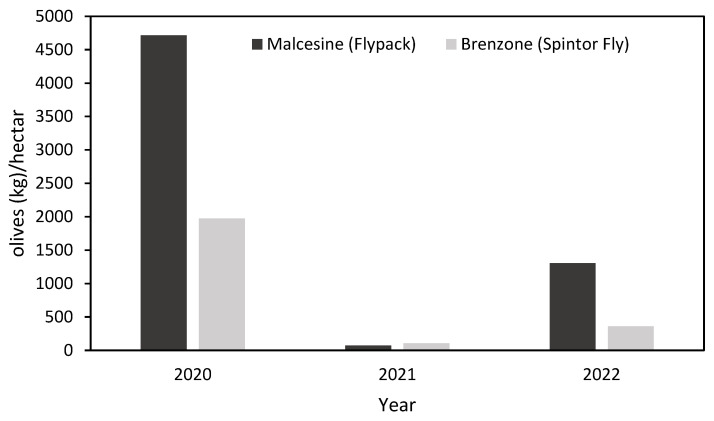
Weight of olives harvested per hectare at the sites where the mass trapping (Flypack) and lure-and-kill (Spintor Fly) techniques were tested over three years.

The olive production varied between the three years. In 2020, a high-yield year, harvests reached 4716 kg/ha and 1973 kg/ha at the experimental sites used for testing MT and LK techniques, respectively. In contrast, 2021 was a low-yield year, with production dropping to 72 kg/ha and 108 kg/ha. In 2022, productivity slightly increased, reaching 1304 kg/ha and 363 kg/ha (Figure 3).

## 4. Discussion

This study was conducted in the Lake Garda area, the northernmost boundary of olive cultivation in Europe, which has experienced severe *B. oleae* infestations in recent decades. The presence of numerous olive groves with steep slopes, often surrounded by urban areas, poses challenges for conventional control methods, also for the limited accessibility of agricultural machinery. The lake’s microclimate supports both olive cultivation and olive fly development, although yields vary considerably between years [19,25], as also observed during the time frame considered. While productivity can drop to zero in certain seasons, the proximate causes remain unclear. In this context, the aim of this study was to evaluate the effectiveness of sustainable control techniques. We found that the efficacy of the AK techniques varied across years and that fruit infestation was associated with olive production levels. During the three monitoring seasons, infestation and yield trends were similar at both experimental sites, despite differences in overall productivity. In 2020, infestation rates remained below 5% throughout most sampling dates, and were associated with high olive production (Figure 3). In contrast, in 2021, infestation reached up to 100% between plots and sampling rounds, coinciding with a low-yield year. In 2022, infestation rates were around 20%, corresponding to intermediate production levels. These findings are consistent with previous studies that linked olive infestation with olive fruit yield of the orchard [11,26], with years of high fruit yield coinciding with low infestation levels, alternating with years of low fruit yield and high infestation levels. From this perspective, the quantity of olives available in a given year is a key factor affecting infestation rates. Years of abundant production may reduce the detectability of observable damage due to the high availability of resources for OLF oviposition. Conversely, during low production years, the limited number of olives could lead to high infestation rates and possibly increased fly movement due to the scarcity of oviposition sites. For instance, in 2021, OLF densities in control plots were high, while densities in plots treated with the AK techniques were lower and comparable to those in other years. Despite lower densities, infestation rates remained elevated, indicating that trap catches do not always correlate with infestation levels [26].

The olive fly population dynamics are primarily influenced by climatic conditions, while infestation rates are more closely linked to olive production. Favorable climatic conditions can sustain large populations, but in low-production years, even small populations may result in high infestation due to increased oviposition pressure per fruit. As the AK techniques were applied in two olive groves, microclimatic conditions and differences in olive fly population densities among nearby groves could also have influenced the results. When assessing the final quantitative damage, several factors should be taken into account, including fruit variety, fruit developmental stage, and harvest time.

Notably, the number of days with temperatures exceeding 30 °C, conditions that could limit OLF development and activity [25], was similar across the three monitoring seasons with 28 days in 2020, 27 days in 2021, and 48 days in 2022 (www.arpa.veneto.it). Therefore, high summer temperatures should not be considered a limiting factor for OLF development in this study, particularly since trap catches remained high even during the hottest year (2022). Other potential factors to consider include humidity and precipitation levels, which may influence insect movement, development, and fruit suitability [27,28]. However, the number of rainy days was comparable across years (19 days on average from July to September), suggesting a limited role of precipitation variability in explaining differences in infestation levels. Nevertheless, olive fly infestations should be closely monitored under climate warming scenarios, as rising temperatures—combined with the favorable microclimate created by the lake—may alter adult phenology in spring and extend the oviposition period in autumn [29].

The quantity of olives produced in a given year, can also impact OLF density in the following season. In high-yield years (e.g., 2020), mechanical harvesting may leave a significant number of olives on the trees, providing suitable oviposition sites the following spring and leading to an early population increase [30,31]. Similarly, in years of low or no production, the lack of harvesting could leave a reservoir of olives for overwintering populations, but the quantity may be insufficient to sustain a significant increase in OLF numbers.

In conclusion, Spintor Fly was effective over the three years, but its low persistence and the risk of wash-off from rainfall required repeated applications. In contrast, Flypack proved to be a less effective alternative, despite offering ease of use and long-lasting action [18,32]. The AK techniques tested, particularly LK, can help maintain low OLF densities but are not sufficient to keep fruit infestation at acceptable levels in years of low olive production. In that case, the application of control methods is not economically viable, as the production gain does not cover the cost of the control methods. Therefore, under such conditions, the adoption of additional control measures is not economically justified. However, in years of medium to high olive production, the simultaneous and early application of AK techniques could help maintain low olive fly population levels throughout the year. Moreover, a wide-area protection strategy should be implemented to prevent re-infestation from adjacent groves [33].

## Figures and Tables

**Table 1:** Results of the linear mixed effects models (Type II Wald χ^2^ tests) testing the effects of treatment (a) Flypack (vs. control) in one grove and (b) Spintor Fly (vs. control) in the other grove and sampling year on % of infested olives. The models included sampling date as random factor.

Explanatory Variables	χ^2^	df	*p*-Value
(a) Flypack			
Treatment	1.121	1	0.289
Year	52.999	2	<0.001
Treatment × Year	7.128	2	0.028
(b) Spintor Fly			
Treatment	279.993	1	<0.001
Year	36.907	2	<0.001
Treatment × Year	60.522	2	<0.001

## Data Availability

The raw data supporting the conclusions of this article will be made available by the authors on request.

## Supplementary Materials

The following supporting information can be downloaded at: https://www.mdpi.com/article/10.3390/insects16111161/s1, Table S1: Infestation percentage recorded at each survey date in two sites where the mass trapping (Flypack) and lure-and-kill (Spintor Fly) techniques were individually tested over three years.

## References

[1] Manousis T., Moore N.F. (1987). Control of *Dacus oleae*, a major pest of olives. Int. J. Trop. Insect Sci..

[2] Medjkouh L., Tamendjari A., Keciri S., Santos J., Nunes M.A., Oliveira M.B.P.P. (2016). The effect of the olive fruit fly (*Bactrocera oleae*) on quality parameters, and antioxidant and antibacterial activities of olive oil. Food Funct..

[3] Gonçalves M.F., Santos S.A., Torres L.M. (2012). Efficacy of spinosad bait sprays to control *Bactrocera oleae* and impact on non-target arthropods. Phytoparasitica.

[4] Pinheiro L.A., Dáder B., Wanumen A.C., Pereira J.A., Santos S.A., Medina P. (2020). Side effects of pesticides on the olive fruit fly parasitoid *Psyttalia concolor* (Szépligeti): A Review. Agronomy.

[5] Zhou Y., Zhao W., Lai Y., Zhang B., Zhang D. (2020). Edible plant oil: Global status, health issues, and perspectives. Front. Plant Sci..

[6] Vizzarri V., Lombardo L., Novellis C., Rizzo P., Pellegrino M., Cruceli G., Godino G., Zaffina F., Ienco A. (2023). Testing the Single and Combined Effect of Kaolin and Spinosad against *Bactrocera oleae* and Its Natural Antagonist Insects in an Organic Olive Grove. Life.

[7] Checchia I., Perin C., Mori N., Mazzon L. (2022). Oviposition deterrent activity of fungicides and low-risk substances for the integrated management of the olive fruit fly *Bactrocera oleae* (Diptera, Tephritidae). Insects.

[8] Kapranas A., Collatz J., Michaelakis A., Milonas P. (2022). Review of the role of sterile insect technique within biologically-based pest control–An appraisal of existing regulatory frameworks. Entomol. Exp. Appl..

[9] El-Sayed A.M., Suckling D.M., Byers J.A., Jang E.B., Wearing C.H. (2009). Potential of “lure and kill” in long-term pest management and eradication of invasive species. J. Econ. Entomol..

[10] Noce M.E., Belfiore T., Scalercio S., Vizzarri V., Iannotta N. (2009). Efficacy of new mass-trapping devices against *Bactrocera oleae* (Diptera tephritidae) for minimizing pesticide input in agroecosystems. J. Environ. Sci. Health B.

[11] Broumas T., Haniotakis G., Liaropoulos C., Tomazou T., Ragoussis N. (2002). The efficacy of an improved form of the mass-trapping method, for the control of the olive fruit fly, *Bactrocera oleae* (Gmelin) (Dipt., Tephritidae): Pilot-scale feasibility studies. J. Appl. Entomol..

[12] Yasin S., Rempoulakis P., Nemny-Lavy E., Levi-Zada A., Tsukada M., Papadopoulos N.T., Nestel D. (2014). Assessment of lure and kill and mass-trapping methods against the olive fly, *Bactrocera oleae* (Rossi), in desert-like environments in the Eastern Mediterranean. Crop Prot..

[13] López S., Acín P., Gómez-Zubiaur A., Corbella-Martorell C., Quero C. (2024). A shift in the paradigm? A male-specific lactone increases the response of both sexes of the olive fruit fly *Bactrocera oleae* to the food lure ammonium bicarbonate. J. Pest Sci..

[14] Petacchi R., Rizzi I., Guidotti D. (2003). The ‘lure and kill’ technique in *Bactrocera oleae* (Gmel.) control: Effectiveness indices and suitability of the technique in area-wide experimental trials. Int. J. Pest Manag..

[15] Broumas T., Haniotakis G.E. (1994). Comparative field studies of various traps and attractants of the olive fruit fly, *Bactrocera oleae*. Entomol. Exp. Appl..

[16] Bjeliš M. Control of olive fruit fly-*Bactrocera oleae* Rossi (Diptera, Tephritidae) by mass trapping and bait sprays methods in Dalmatia. Proceedings of the 11th Slovenian Conference of Plant Protection.

[17] Broumas T., Haniotakis G. Further studies on the control of the olive fruit fly by mass trapping. Proceedings of the Second International Symposium.

[18] Mucci M., Baldessari M., Michelotti F., Chiesa S.G., Angeli G. Attract and Kill for the control of olive fruit fly in Alto Garda, Trentino. Proceedings of the IOBC-WPRS Working Groups.

[19] Gulotta T.M., Mondello G., Salomone R., Primerano P., Saija G. (2025). Addressing geographical variability in Life Cycle Inventory data: The case of Italian olive production. Int. J. Life Cycle Assess..

[20] Istituto di Servizi per il Mercato Agricolo Alimentare—ISMEA. https://www.ismeamercati.it/olio-oliva.

[21] Sanz-Cortés F., Martinez C.J., Badenes M.L., Bleiholder H., Hack H., Llácer G., Meier U. (2002). Phenological growth stages of olive trees (*Olea europaea*). Ann. Appl. Biol..

[22] Girolami V. (1979). Studies on the biology and population ecology of *Dacus oleae* (Gmelin). 1. Influence of environmental abiotic factors on the adult and on the immature stages. Redia.

[23] Marchini D., Petacchi R., Marchi S. (2017). *Bactrocera oleae* reproductive biology: New evidence on wintering wild populations in olive groves of Tuscany (Italy). Bull. Insectol..

[24] R Core Team (2024). R: A Language and Environment for Statistical Computing. https://www.r-project.org/.

[25] Gutierrez A.P., Ponti L., Cossu Q.A. (2009). Effects of climate warming on olive and olive fly (*Bactrocera oleae* (Gmelin)) in California and Italy. Clim. Chang..

[26] Varikou K., Garantonakis N., Birouraki A., Ioannou A., Kapogia E. (2016). Improvement of bait sprays for the control of *Bactrocera oleae* (Diptera: Tephritidae). Crop Prot..

[27] Yokoyama V.Y. (2012). Olive fruit fly (Diptera: Tephritidae) in California: Longevity, oviposition, and development in canning olives in the laboratory and greenhouse. J. Econ. Entomol..

[28] Katsikogiannis G., Kavroudakis D., Tscheulin T., Kizos T. (2023). Population dynamics of the olive fly, *Bactrocera oleae* (Diptera: Tephritidae), are influenced by different climates, seasons, and pest management. Sustainability.

[29] Rondoni G., Mattioli E., Giannuzzi V.A., Chierici E., Betti A., Natale G., Petacchi R., Famiani F., Natale A., Conti E. (2024). Evaluation of the effect of agroclimatic variables on the probability and timing of olive fruit fly attack. Front. Plant Sci..

[30] Delrio G., Lentini A. (2016). Dinamica e fattori di regolazione delle popolazioni della mosca delle olive. Atti Accad. Naz. Ital. Entomol..

[31] Mazomenos B.E., Pantazi-Mazomenou A., Stefanou D. (2002). Attract and kill of the olive fruit fly *Bactrocera oleae* in Greece as a part of an integrated control system. IOBC WPRS Bull..

[32] Lentini A., Delrio G., Foxi C. (2005). Experiments for the control of olive fly in organic agriculture. IOBC WPRS Bull..

[33] Stavrianakis G., Sentas E., Zafeirelli S., Tscheulin T., Kizos T. (2025). Utilizing olive fly ecology towards sustainable pest management. Biology.

