# Supplementary material for: Efficacy of Attract-and-Kill Techniques in Controlling Bactrocera oleae (Diptera: Tephritidae) in a Highly Variable Olive Production Scenario
Source: Insects. 2025 Nov 13;16(11):1161. doi: 10.3390/insects16111161 (PMC12653673; doi:10.3390/insects16111161)
Supplement: Supplementary file 1 [file insects-16-01161-s001.zip › insects-3887246-supplementary.pdf]

Table S1. Infestation percentage recorded at each survey date in the two sites where the mass trapping (Flypack) and lure-and-kill (Spintor Fly) techniques were individually tested over three years.

**Year**	**Date**	**Flypack**	**Control**	**Spintor Fly**	**Control**
2020	23 July	0	1.4	0.6	1.2
	03 August	0.2	0	0	0
	19 August	0.3	2.5	1.0	0.7
	03 Septmeber	0.6	1.6	1.1	2.8
	23 September	0.4	2.6	0.7	1.6
	06 October	3	4.2	1.1	0
	20 October	15.8	12.6	3.8	13
2021	03 August	53.2	47	12	7
	19 August	94.4	96.2	38.5	30.1
	03 Septmeber	96	99	27	55.6
	23 September	95.6	77.2	14	88.5
	06 October	100	100	47.8	97.2
2022	03 August	2.4	0.4	1.6	0.4
	19 August	1.6	1.6	2.6	2.4
	03 Septmeber	1	1.6	1	1.6
	23 September	14.1	18.4	3.2	11.4
	06 October	23.4	21.8	6.1	12.2
	20 October	29.4	36.5	10.7	22.7
